# Three-dimensional ultrastructural characterization of *Drosophila melanogaster* hygrosensilla across humidity conditions

**DOI:** 10.1371/journal.pone.0314841

**Published:** 2025-09-29

**Authors:** Ganesh Giri, Anders Enjin

**Affiliations:** Department of Experimental Medical Science, Lund University, Lund, Sweden; Central Food Technological Research Institute CSIR, INDIA

## Abstract

Understanding how organisms detect environmental humidity remains a fundamental problem in sensory biology. While specialised sensory neurons in insect antennae can detect changes in humidity, the mechanism underlying this ability is not fully understood. Here, we present an integrated approach combining precise humidity control, rapid cryo-preservation, and serial block-face scanning electron microscopy (SBF-SEM) to investigate the ultrastructure of hygrosensilla in the vinegar fly *Drosophila melanogaster*. We developed a deep learning-based segmentation pipeline to analyse three-dimensional structural features of sensilla exposed to different humidity conditions at stable temperature. Our analysis reveals consistent differences in sensilla width between high and low humidity conditions across all chambers of the sacculus. Additionally, we identified chamber-specific patterns in sensilla tapering, indicating specialised structural adaptations across different sensilla populations. The observed structural differences suggest a potential role for mechanical transduction in humidity sensing. This study establishes a technical framework for high-resolution analysis of sensory organs while providing new insights into the structural basis of humidity detection. Our findings advance our understanding of how specialised sensory organs might transduce environmental signals into neural responses.

## Introduction

Humidity is a fundamental environmental factor that influences the ecology and behaviour of terrestrial organisms [1–3]. The ability to sense and respond to changes in ambient moisture, known as hygrosensation, is crucial for a wide range of species. Small ectotherms, such as insects, are particularly sensitive to humidity due to their high surface-area-to-volume ratio, which makes them vulnerable to water loss [4,5]. Numerous species have evolved to utilize local variations in humidity as vital cues for essential behaviours such as foraging, reproduction, and habitat selection [6–13]. Despite its importance in the biology of diverse organisms, our understanding of the mechanisms underlying hygrosensory transduction remains incomplete, presenting a significant gap in our knowledge of sensory biology.

In insects, humidity is sensed via specialised sensory hairs called hygrosensilla, typically found on the antennae [14,15]. These hygrosensilla are often located in protected areas, such as invaginations or grooves, shielding them from wind and mechanical disturbance, presumably to provide a more accurate reading of environmental humidity. For example, in the vinegar fly *Drosophila melanogaster*, the hygrosensilla are located in Chambers 1 and 2 of a relatively large invagination on the posterior side of the antenna called the sacculus, while Chamber 3 contains olfactory sensilla [16–18]. The typical hygrosensillum houses a triad of sensory humidity receptor neurons (HRNs): a moist neuron that increases its firing rate in response to rising humidity, a dry neuron with an inverted response profile, and a hygrocool neuron sensitive to cooling [19,20].

The unique features of hygrosensilla present a challenge to understanding hygrosensory transduction. These features include their poreless structure, protected location, and composition of both hygrosensory and thermosensory neurons. Furthermore, the ambiguous nature of water vapour as a ‘ligand’ for a receptor submerged in a water-based solution (lymph) complicates this understanding. Three main hypotheses have been proposed to explain the mechanism of hygrosensation: mechanosensation, osmosensation, or thermosensation [21,22]. The mechanosensation model suggests that HRNs detect structural changes in hygrosensilla through mechanically-gated channels. The osmosensation hypothesis proposes that HRNs detect changes in ion concentration in the sensillum lymph due to water evaporation. The thermosensation model suggests that HRNs detect the rate of cooling caused by evaporative cooling. Electrophysiological studies have revealed important insights into how the HRNs might work together to detect changes in humidity. Recordings from the moist and dry cells show a double dependency on both temperature and humidity that cannot be reduced to a common parameter such as evaporative cooling [22]. For example, in cockroach hygroreceptors, when water vapour pressure is oscillated at different temperature levels, the discharge rate of moist cells increases with rising vapour pressure while dry cells show the opposite pattern [22]. Importantly, the higher the temperature of these oscillating changes in vapour pressure, the stronger the oscillating responses of both moist and dry cells. The fact that both cells’ responses are modulated by humidity and temperature suggests that evaporative cooling alone cannot fully explain hygrosensation. Moreover, when the water vapour content of the air remains constant, but temperature rises, relative humidity (RH) decreases, yet impulse frequency in both moist and dry cells increase with rising temperature [22]. This positive temperature coefficient of the hygroreceptors’ responses to changes in RH contradicts a purely mechanical transduction model. Together, these findings indicate that hygroreceptors likely use multiple mechanisms, combining temperature, osmotic and/or mechanical inputs in ways that cannot be explained by any single transduction model. Furthermore, studies in *C. elegans* have shown that hygrosensation requires a combination of distinct mechanosensitive and thermosensitive pathways, suggesting this dual-input strategy may be evolutionarily conserved across species that lack specialised hygroreceptors [23]. Similarly, in human wetness perception, both mechanical pressure and cold sensation through TRPM8 channels are required [24].

Several studies have suggested that mechanosensation is a key feature of hygrosensation also in insects, however, demonstrating this morphologically has proved difficult [25–27]. Studies on the domestic silk moth *Bombyx mori* found humidity-induced morphological changes, but only after extended periods of dry and moist adaptation, preventing insight into immediate structural responses [28]. A different approach using in situ atomic force microscopy on honey bee *Apis mellifera* hygrosensilla revealed only limited lateral structural changes, possibly due to technical constraints in detecting fine-scale alterations [29]. Here, we use rapid plunge-freezing combined with serial block-face scanning electron microscopy (SBF-SEM) to investigate rapid structural responses of *D. melanogaster* hygrosensilla to humidity change. This approach allows us to capture and analyse three-dimensional structural changes across the entire sensillum, revealing consistent differences in sensilla dimensions between high and low humidity conditions and providing new insights into how these specialised sensory organs might transduce environmental signals into neural responses.

## Materials and methods

### Sample preparation

Female *w*^*1118*^ flies (standard laboratory control strain; Bloomington ID: 5905), aged 7–14 days and with intact appendages and antennae, were selected for the experiment. Flies were reared in vials containing cornmeal agar medium maintained at 25°C under 12-hour dark/light cycle within an incubator. Inside home vials, humidity levels ranged from 90% RH near the food to 70% RH farthest away from the food. Only females were used to eliminate potential sex-specific differences in sensilla morphology. A total of one fly per humidity condition was processed for SBF-SEM analysis.

**Fig 1:**
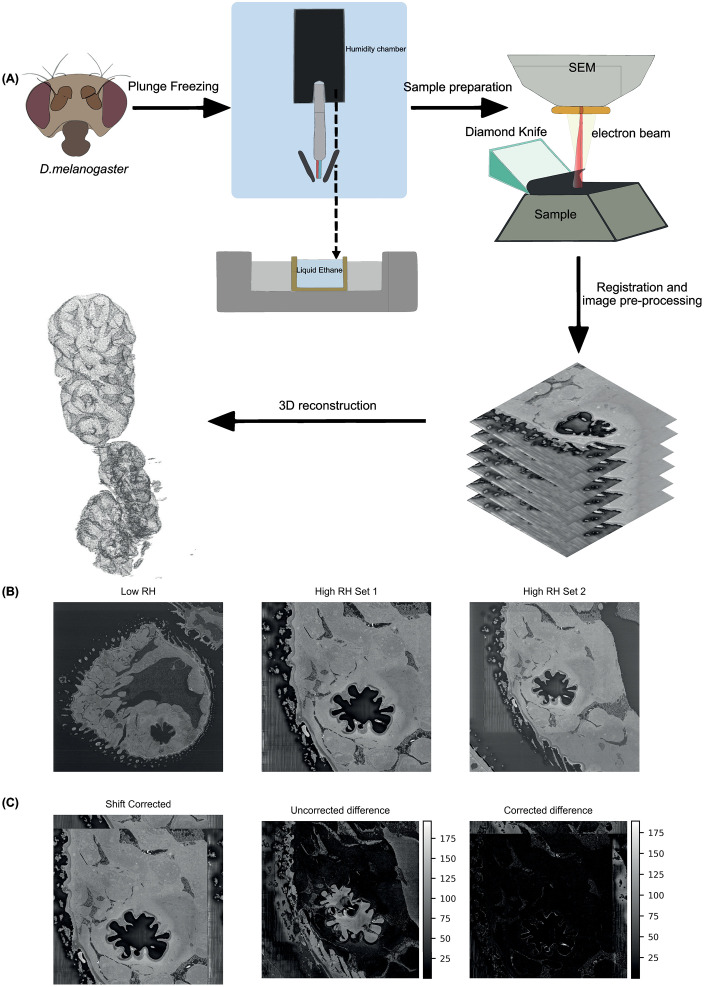
Workflow for sample preparation, imaging, and segmentation of *D. melanogaster* sacculus under different humidity conditions. (A) Schematic of the plunge-freezing protocol: flies were placed in a humidity-controlled chamber (26% or 80% RH, 22°C) for 45 seconds, then plunge-frozen in liquid ethane to preserve their structural state. The sample was stained, embedded in epoxy resin, and trimmed to expose the antenna, which was imaged in its entirety using SBF-SEM. Segmentation of the sacculus from these images was achieved using a U-Net model, enabling three-dimensional reconstruction. (B) Sample images acquired at low RH (Left) and high RH (Center and Right). (C) High RH sample images (Set 2) adjusted to match Set 1 resolution, with difference images showing Set 1 vs. Set 2 before (Center) and after (Right) shift correction and resolution correction.

To preserve the flies under a specific humidity condition, they were attached to forceps positioned within the humidity-controlled chamber of a Vitrobot Mark IV system (FEI, Denmark) and were held at the desired humidity level for approximately 45 seconds before being plunged into liquid ethane. The setup included a crucible on a platform beneath the chamber, with liquid nitrogen in the outer cavity and liquid ethane in the inner cavity. This rapid freezing process ensured that the fly was preserved under the designated humidity levels (Fig 1A). Two humidity conditions were used: high humidity at 80% RH and low humidity at 26% RH, both maintained at 22°C and controlled by the Vitrobot Mark IV system.

Following plunge-freezing, samples were transferred to a freeze substitution medium containing 0.1% tannic acid and 0.5% glutaraldehyde in acetone and incubated at −90°C for 96 hours in a Leica AFS2 (Leica, Denmark). Samples were then washed four times with anhydrous acetone to remove the initial freeze substitution medium. The acetone wash was replaced with a fresh solution of 2% osmium tetroxide (OsO₄) in anhydrous acetone. The freeze substitution process was continued with this OsO₄ solution for an additional 28 hours. Osmium tetroxide was used to stain the samples, providing contrast for electron microscopy by binding to lipids and preserving cellular structures. After staining, the temperature was gradually raised over 14 hours to −30°C, where the samples were held for 16 hours. To remove the OsO₄ and any remaining residues, the samples were washed four more times with anhydrous acetone. The temperature was then further increased over 4 hours to 1°C.

Dehydration was performed through a graded ethanol and acetone series, with each step lasting 10 minutes. The sequence included 70% ethanol on ice, 90% ethanol on ice, two changes of 100% ethanol on ice, followed by ice-cold 100% acetone at room temperature, and finally, 100% acetone at room temperature. After dehydration, the samples were embedded in Epon for further processing.

### Imaging

The embedded resin block was positioned and trimmed to achieve an optimal angle for acquiring antennal sections. SBF-SEM was performed on the trimmed samples using the Teneo VolumeScope (FEI, Denmark). For the sample prepared at 80% RH (high RH dataset), a total field of view of 42.5 µm × 42.5 µm, with a total depth of 14.46 µm was achieved with the imaging resolution of 10.3760 nm × 10.3760 nm in the XY plane and 30 nm in the Z axis (high RH Set 1), with the sample sliced into 482 sections of 30 nm thickness each. Beam parameters included an energy of 2.28 keV, an electron dose of 69.57 e/nm², a beam current of 400 pA, and a dwell time of 3 µs. Imaging was conducted under low vacuum conditions, using a VsGAD detector with line and frame integration set to 1. The tile resolution was 4,096 × 4,096 pixels. Multi-Energy Deconvolution (MED) was applied to enhance contrast by combining images captured at different energy levels, effectively reducing noise and improving structural detail. Following an interruption in the imaging process, the sample was repositioned, and imaging was resumed. This led to a change in imaging parameters. The field of view was expanded to 60.0 µm × 60.0 µm, covering a total depth of 30.03 µm with a resolution of 14.6484 nm × 14.6484 nm in the XY plane (high RH Set 2). The resolution along the Z axis remained at 30 nm. A total of 1,001 slices were acquired under similar beam conditions, with an electron dose of 34.91 e/nm². The same detector and imaging settings were used as before.

For the sample prepared at 26% RH (low RH dataset), the imaging conditions were similar without any interruptions during image acquisition. A field of view of 111.0 µm × 111.0 µm and a depth of 104.16 µm was achieved with a resolution of 10.839 × 10.839 nm in the XY plane and 30 nm in the Z plane. A total of 3472 slices were collected with an energy of 2.27 keV and an electron dose of 10.62 e/nm² with a dwell time of 1 µs.

### Image rescaling and alignment for high RH dataset

To align and analyse the images from the high-humidity dataset (80% RH), it was necessary to account for the differences in resolution between two sets of images obtained during the experiment (high RH Set 1 and high RH Set 2). The first set of images had a pixel size of 10.3760 nm × 10.3760 nm in the XY plane, while the second set had a larger pixel size of 14.6484 nm × 14.6484 nm. To account for this discrepancy, scale factors were calculated based on the pixel size ratio between the two sets. The images from the second set were rescaled according to the scaling factor to obtain a resolution of 10.3760 nm × 10.3760 nm in the XY plane. Rescaling was done with the help of rescale function from the skimage library, with anti-aliasing applied to minimize artifacts [30]. Following rescaling, the images from the second set were cropped or padded to match the exact dimensions of the first set.

For alignment, the last image from the high RH Set 1 and the first rescaled image from the high RH Set 2 were compared using phase cross-correlation, implemented via phase_cross_correlation function from the skimage library. This method estimated the translational shift between the two images, which was then applied to the entire second set using a Fourier shift-based method, ensuring subpixel accuracy. After applying the shift, the images were normalized to be in the range of 0–255 to maintain consistent intensity levels throughout the dataset. The final aligned and normalized images were saved for further analysis.

### Generating image labels

A custom Python script was used to randomly select 337 images from both the High and low RH datasets, ensuring an equal proportion of images from each condition. The sacculus outlines were manually traced using the drawing tools in IMOD software [31]. These traced outlines were then converted into a binary label stack using the imodmop function to generate binary labels, with the mask value set to 1.

### Data augmentation

To increase the diversity of training data for the machine learning model, data augmentation was performed on the labelled images. For each labelled image, four new augmented versions were generated: vertically flipped, horizontally flipped, and rotated by 45 and 130 degrees. The augmented images and their corresponding labels were saved in separate directories for further use in training the model.

### Segmentation

A deep learning-based approach was implemented using a U-Net architecture to segment the sacculus from the acquired images [32]. The images and their corresponding labels were resized to a dimension of 256 × 256 pixels and then normalized to have pixel values between 0 and 1. The pre-processed data was then split into training, test and validation sets in the ratio of 90:5:5.

The U-Net model was compiled using Adam optimizer with binary cross entropy as the loss function. Accuracy and Intersection over union (IoU) were used as metrics to evaluate the training of the model. Since the region of interest corresponding to the sacculus was much smaller in area compared to the background, IoU metric was given more importance compared to the accuracy metric during the evaluation of the model.

Two callback functions from the TensorFlow library were implemented to enhance the performance of the trained model [33]. The first was ModelCheckpoint, which saved the model with the best IoU score during the training to ensure that the model with the highest overlap between the predicted mask and ground truth was saved. The other callback being ReduceLROnPlateau which dynamically reduced the learning rate whenever the validation loss plateaued, aiding in more effective convergence of the model. Using the trained model, predicted labels segmenting the sacculus were generated for both the low and high RH datasets. The predicted labels had a size of 256 × 256 pixels.

### Generating three-dimesional structure of the sacculus

Contours were extracted from the predicted labels using contour detection function from the the OpenCV library with RETR_TREE as the retrieval mode [34]. This allowed for the detection of all the nested contours within the predicted labels. Detected contours were stacked and assigned a Z coordinate according to its position in the image sequence. The contour stack was then rescaled to match the original image dimension and to account for the physical resolution of the data set. The scaled contour points were then used to generate a point cloud which visualized the 3-dimensional structure of the segmented sacculus.

### Isolation and alignment of the sensilla

The point cloud was visualized using the Open3D library, which facilitated the identification and localization of individual sensilla [35]. The identified sensilla were then manually cropped using the draw_geometries_with_editing function from Open3D.

The cropped sensilla varied in orientation depending on their position within the sacculus, which made measuring their dimensions challenging. Therefore, orienting them along a specific axis was important. To standardize orientation, we selected one representative sensillum from each chamber (Chambers 1 and 3) and centered its point cloud at the origin by subtracting the centroid coordinates from each point. Principal Component Analysis (PCA) was then applied to this centered point cloud to identify its principal axes. In sensilla with a large height-to-width ratio, such as those in chambers 1 and 3, the axis with the greatest variance corresponded to the sensilla’s length. This axis was designated as the principal axis. The angle between this principal axis and the Z axis was then calculated, and a rotation was applied to orient the sensillum along the Z axis. This oriented sensillum served as the target for aligning the remaining sensilla within the chamber. A RANSAC algorithm [36] was implemented to perform global registration, aligning each remaining sensilla’s point cloud with that of the target. First, the point clouds were downsampled, and then a global registration process was used to compute the optimal transformation matrix, achieving the best alignment with the target.

However, in the case of Chamber 2, the PCA analysis did not yield the correct orientation, as the chamber’s length and width were similar, causing the axis of maximum variance not to correspond with the length of the sensilla. To address this, a conical point cloud matching the dimensions of the sensilla in Chamber 2 was created and oriented along the Z axis. This synthetic point cloud served as the target for alignment. Using a RANSAC-based approach, the sensilla in Chamber 2 were aligned to the target point cloud, ensuring accurate and consistent orientation for all sensilla within the chamber.

### Width measurement

The aligned point clouds were converted into a three-dimensional mesh using a Delaunay triangulation approach. The meshes were then sliced at different heights to generate 2D cross-sectional slices. For each slice, the width of the sensilla was determined by calculating the maximum distance between the extreme points along the XY plane. This effectively captured the widest span of the structure at that specific height. This process was repeated across multiple slices, providing a width profile along the height of the sensilla.

Noise in width measurement was reduced by applying a weighted moving average (WMA) filter to the width profile. After smoothing, the width of the corresponding height values were plotted to analyse the sensilla’s width distribution along its length.

### Data analysis

A mixed-effects model from statsmodels was implemented to examine the relationship between sensilla width and height, while accounting for differences between the high and low RH dataset [37]. The model included a fixed effect for the interaction between height and group (humid vs. dry) to capture the influence of environmental conditions on width. Additionally, a random effect for individual sensilla was incorporated to account for inherent variability between sensilla. This random effect allowed the model to adjust for baseline differences in width across individual sensilla, ensuring that the analysis focused on the effects of the humidity conditions rather than on the natural variation between sensilla.

After fitting the model, predictions were generated for the width of sensilla across a range of heights for both groups. The model results were used to calculate 95% confidence intervals around the predicted widths to evaluate the uncertainty in the estimates.

The dimensions of the sensilla were further compared using full width half maximum (FWHM). FWHM for each sensilla was determined by identifying the height of individual sensilla and then locating the corresponding width at half the value of the determined height, thereby providing a standardized method to compare the overall size of the sensilla. To assess differences between groups, the FWHM distributions were compared using a non-parametric Mann-Whitney U test.

## Results

### Structural characterization of sensilla across humidity conditions

The SBF-SEM imaging provided comprehensive volumetric data of the sacculus, covering dimensions of 42.5 × 42.5 × 14.46 µm (high RH Set 1) and 60.0 × 60.0 × 30.03 µm (high RH Set 2) at resolutions of 10.38 and 14.65 nm/pixel respectively (Fig 1A, S1 Fig). For the low humidity condition, we acquired a larger volume of 111.0 × 111.0 × 104.16 µm at 10.84 nm/pixel resolution (Fig 1B, C, S1 Fig). These high-resolution datasets enabled three-dimensional reconstruction of the complete sacculus structure, including all three chambers and their associated sensilla.

**Fig 2:**
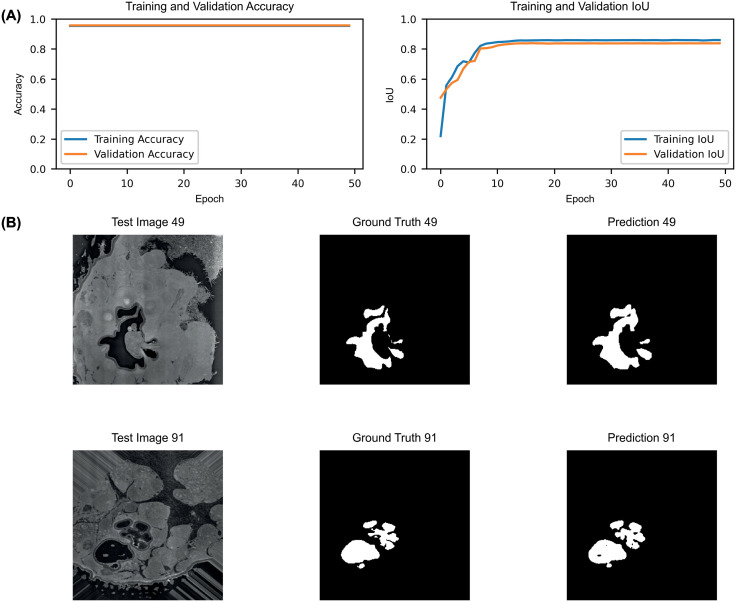
Evaluation and application of the U-Net model for sacculus segmentation. (A) Model performance metrics, showing accuracy (Left) and Intersection over Union (IoU), for both training and validation datasets. (B) Segmentation results using the trained U-Net model on test data, displaying the test input (Left), the corresponding ground truth (Center), and the model’s prediction (Right).

To systematically analyse the structural properties of individual sensilla, we developed a deep learning-based U-Net segmentation model to accurately identify and isolate sensilla from the sacculus. The model achieved high classification accuracy, with a 95% overall accuracy throughout the training phase and an Intersection over Union (IoU) metric of 0.854 (Fig 2A), a robust measure of segmentation performance, particularly in regions with a small region of interest relative to the background. Despite the complex structure of the sacculus and the small size of the sensilla, the model performed effectively, allowing for reliable identification of the sacculus across both the high and low RH datasets (Fig 2B).

**Fig 3:**
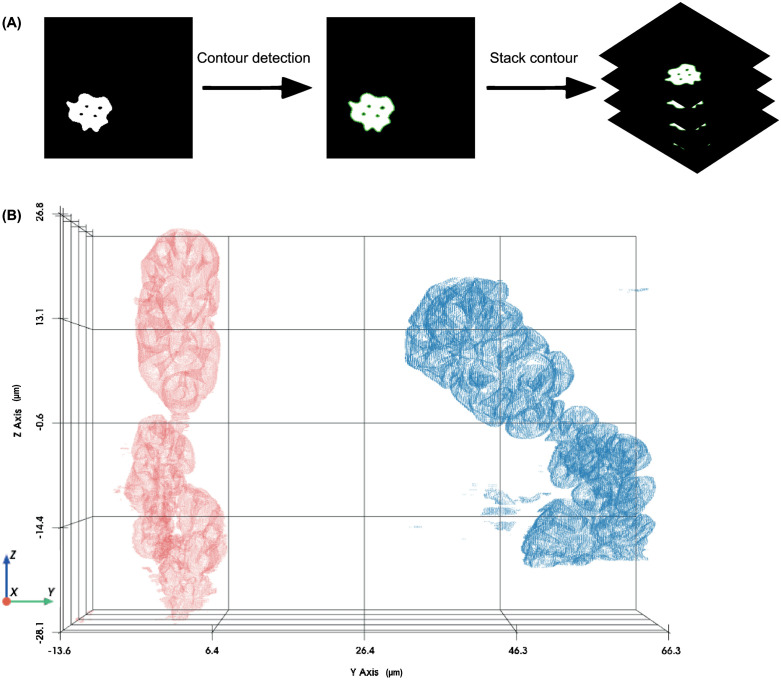
Sacculus structure representation through contour detection and point cloud visualization. (A) Contour detection was performed on each segmented image in the stack. (B) The detected contours were converted into point clouds to visualize the sacculus structure. The red point cloud represents the sacculus from the low humidity (RH) sample, and the blue point cloud represents the sacculus from the high humidity (RH) sample.

**Fig 4:**
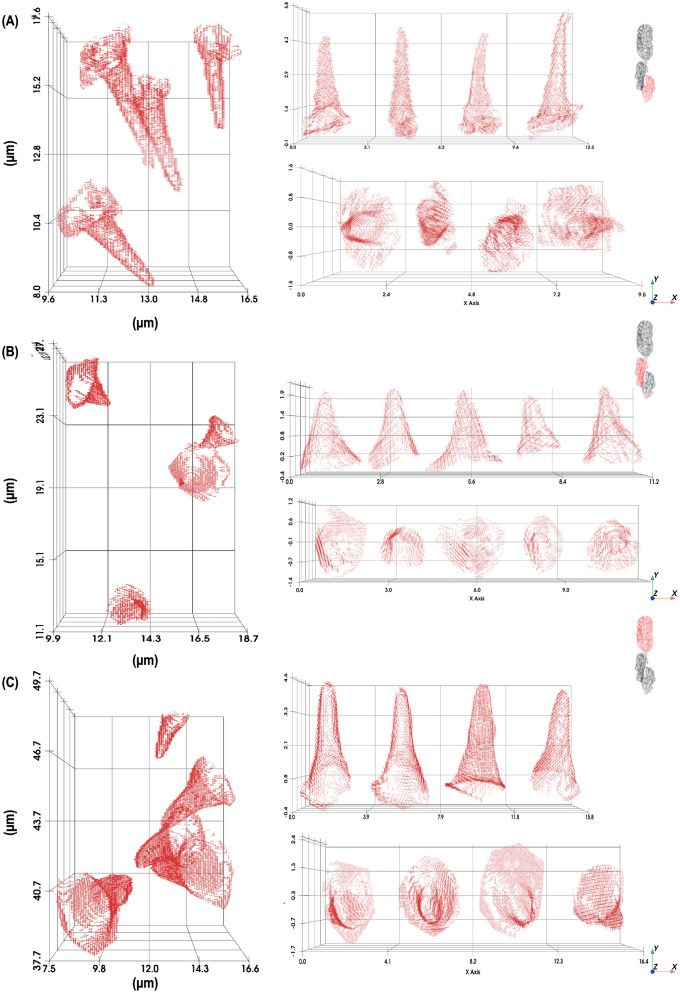
Isolated sensilla from low-humidity sample shown in their original orientation within the sacculus (Left) and after alignment for dimensional measurement (Right). (A) Chamber 1 (hygrosensilla), (B) Chamber 3 (olfactory sensilla), and (C) Chamber 2 (hygrosensilla). For Chamber 2, the green conical point cloud was used as a reference to align sensilla accurately for measurement.

**Fig 5:**
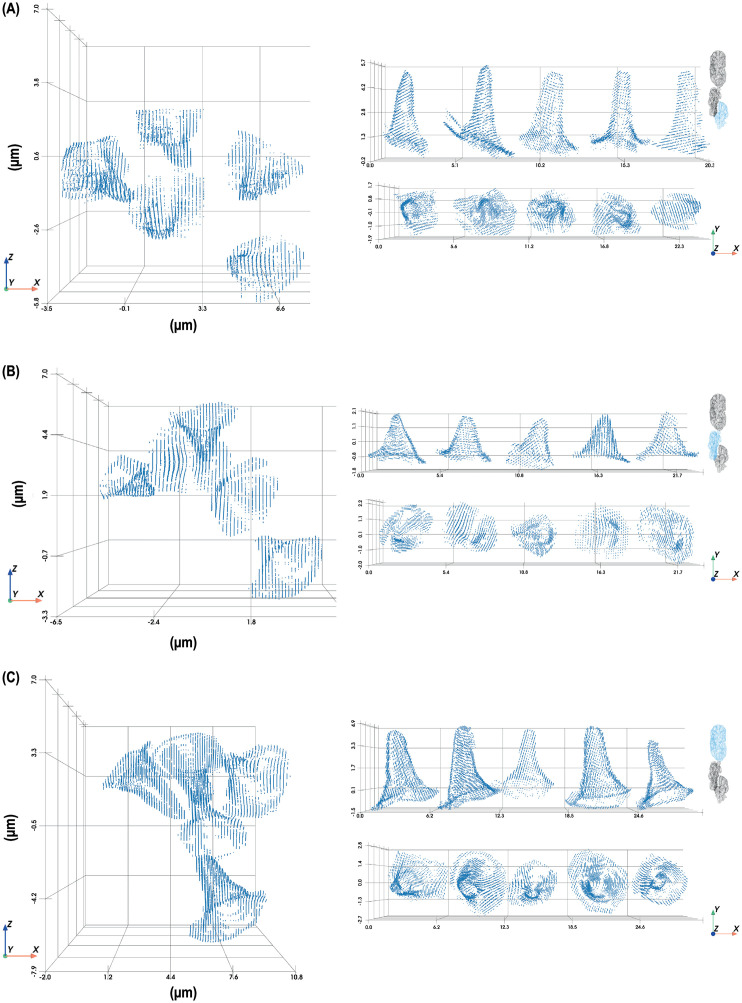
Isolated sensilla from high-humidity sample shown in their original orientation within the sacculus (Left) and after alignment for dimensional measurement (Right). (A) Chamber 1 (hygrosensilla), (B) Chamber 3 (olfactory sensilla), and (C) Chamber 2 (hygrosensilla).

With the contours generated by using the segmented image, we recreated the three-dimensional structure of the sacculus (Fig 3 A, B) and identified distinct sensilla types distributed across the three chambers of the sacculus. The hygrosensilla in chamber 1 appeared elongated and slender with consistent tapering, while hygrosensilla in chamber 2 were shorter and wider with less pronounced tapering. The olfactory sensilla in chamber 3 were elongated but showed regional variation in width within the chamber (Figs 4 and 5).

### Quantitative analysis of sensilla width and height

**Fig 6:**
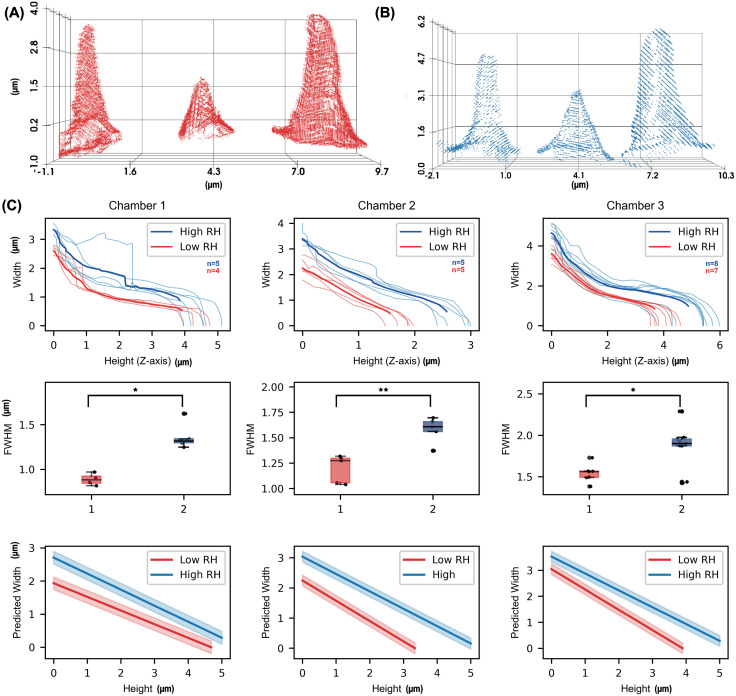
Structural comparison of *D. melanogaster* sensilla across humidity conditions. (A) Sensilla point clouds from low humidity (26% RH) and (B) high humidity (80% RH) samples, oriented and aligned for Chambers 1, 2, and 3 from left to right. (C) Top: Length vs. width profiles for each identified sensillum in Chamber 1 (Left), Chamber 2 (Center), and Chamber 3 (Right), with low humidity samples in red and high humidity samples in blue; darker curves represent the mean length vs. width profiles for each condition. Middle: Full width at half maximum (FWHM) values for each identified sensillum, calculated by determining the height of each sensillum and locating the corresponding width at half this height; statistically significant differences (Mann Whitney U test, * p < 0.05, ** p < 0.01, *** p < 0.001) are observed between high and low RH conditions across all chambers. Bottom: Mixed-effects model of the length vs. width profiles. Solid lines represent calculated profiles, with shaded areas indicating the 95% confidence intervals. Notably, in Chamber 1, high humidity sensilla display a steeper taper (slope: −0.472) compared to low humidity (slope: −0.403). In contrast, sensilla in Chambers 2 and 3 exhibit a steeper taper under low humidity (slopes: −1.120 and −0.652, respectively) than under high humidity (slopes: −0.959 and −0.541).

In the high humidity dataset (80% RH), the average width of the sensilla in Chamber 1 was 3.36 µm with a standard deviation of 0.13 µm. This width was 0.77 µm greater than the mean sensilla width in the low RH dataset (26% RH), where the average width measured 2.59 µm. A similar pattern was observed in chambers 2 and 3, where sensilla in the high humidity samples consistently exhibited wider dimensions than their counterparts in the low humidity condition (Fig 6A, B).

The most pronounced difference in the magnitude of median full-width at half maximum (FWHM) was observed in Chamber 1, with a 0.4 µm increase in sensilla width in the high RH dataset compared to the low RH dataset (p < 0.05). In chambers 2 and 3, the magnitude of this difference was slightly less pronounced but remained statistically significant, with the high humidity sensilla being 0.33 µm wider in both chambers (p < 0.01 for chamber 2 and p < 0.05 for chamber 3) (Fig 6C).

### Height-width relationship across humidity conditions

To further explore the relationship between sensilla width and height under different humidity conditions, we employed a mixed-effects model, which allowed us to account for the inherent variability between individual sensilla while analysing the effects of humidity. The model revealed significant differences in the rate of decline in sensilla width with increasing height between the high and low humidity conditions.

In Chamber 1, sensilla in the high humidity condition exhibited a steeper decline in width with increasing height, with a slope of −0.472 compared to −0.403 in the low humidity condition. This suggests that sensilla under high humidity were not only wider at the base but also showed a sharper tapering toward the tip.

Conversely, in hygrosensilla of Chamber 2 and olfactory sensilla of Chamber 3, the decline in width with height was more pronounced in the low humidity condition. In Chamber 2, the slope was −1.120 under dry conditions compared to −0.959 in the high humidity samples, indicating a steeper reduction in width towards the tip of the sensilla in the low humidity environment. Similarly, in Chamber 3, the slope for the dry condition was −0.652, compared to −0.541 for the humid condition (Fig 6B). These findings suggest that while sensilla in humid conditions are generally wider, they maintain a more gradual taper, potentially facilitating a more nuanced detection of humidity changes across varying sensilla types.

## Discussion

### Structure of sacculus sensilla across humidity conditions

Here, we present a comprehensive ultrastructural analysis of *D. melanogaster* sacculus sensilla using SBF-SEM under controlled environmental conditions. This analysis revealed distinct structural profiles between samples prepared under high humidity (80% RH) and low humidity (26% RH) conditions. Sensilla from the high humidity sample consistently exhibited greater widths compared to those from the low humidity sample across all sacculus chambers. The chamber-specific tapering patterns suggest potential specialization across different sensory populations. Chamber 1 sensilla showed sharper tapering in the high humidity sample, while chambers 2 and 3 exhibited more pronounced tapering in the low humidity sample.. Notably, these structural differences extended beyond hygrosensilla to include olfactory sensilla in chamber 3, indicating that humidity-related structural variation may be a general feature of sacculus sensilla. While the analysis represents static snapshots of likely dynamic responses, and individual variability cannot be fully excluded with single specimens per condition, the consistent patterns are notable. The patterns observed across multiple sensilla within each specimen support a model where structural adaptations contribute to humidity sensitivity.

### Mechanistic basis of humidity detection

Understanding the mechanism of hygrosensory transduction presents unique challenges due to the specialised anatomy of hygrosensilla. Unlike olfactory sensilla that detect airborne molecules through pores in their cuticle, hygrosensilla have a poreless structure and are therefore likely to detect a physical stimulus rather than a chemical [14]. The larger sensilla width observed under high humidity conditions compared to low humidity conditions suggests structural differences may exist between these states. HRNs protrude their sensory cilia into the hygrosensillum and maintain close contact with the cuticular wall [16]. These dimensional changes could potentially activate mechanosensors in the plasma membrane of the HRNs. Transcriptomic analysis of HRNs has revealed distinct molecular signatures that underscore the intimate relationship between sensory neurons and their associated sensilla structures [38]. Dry- and moist-sensing HRNs exhibit differential expression patterns of cuticle-associated proteins potentially forming a link between the membrane of the HRN and the cuticle, with *vermiform* expressed in dry neurons and *fred* expressed in moist neurons [39–41]. Both proteins are integral to chitin metabolism and interaction, suggesting that specialised cuticular architecture may be fundamental to hygrosensory function. Furthermore, these neuronal populations show complementary expression of nicotinic acetylcholine receptor subunits α6 and α7. These receptors are particularly significant as their orthologs have been implicated in mechanotransduction [42,43], potentially indicating a mechanosensory component in hygrosensation.

If humidity-induced expansion and contraction of the sensillum cuticle drives neuronal activation, one would expect the responses to track RH, as any hygroscopic mechanical changes, such as those of a human hair, would correlate with RH [29]. However, electrophysiological studies show that both moist and dry cells display a positive temperature coefficient, their responses to humidity fluctuations increase with rising temperature, contrary to what would be expected from simple RH sensors [21]. This apparent contradiction can be resolved by considering the role of ephaptic coupling between the compartmentalized hygrosensory neurons. Recent work has demonstrated that sensory neurons housed in the same sensillum can inhibit each other through direct electrical field effects mechanisms, without requiring synaptic connections [44–46]. We hypothesize that the hygrocool cell, consistently present alongside the dry and moist cells across insect species, may use ephaptic inhibition to modulate the responses of its neighbouring mechanosensitive neurons. As temperature decreases, the hygrocool cell’s activity increases, leading to stronger ephaptic inhibition of both the dry and moist cells [47]. This explains why their humidity responses are reduced at lower temperatures, despite the mechanical response of the sensillum to RH remaining constant. While speculative, this model provides a functional explanation for the highly conserved grouping in hygrosensilla of these three cell types, the hygrocool cell acts as a temperature-dependent gain control mechanism for the mechanosensitive dry and moist cells through ephaptic coupling.

### Temporal dynamics and spatial organisation of humidity sensing

The structural differences we observe in hygrosensilla must support rapid sensory responses, as flies exhibit swift behavioural reactions to humidity changes [48]. While our SBF-SEM analysis reveals humidity-dependent structural differences, these represent fixed structural states of what is likely a highly dynamic process occurring on a millisecond time scale. The ability to capture these rapid structural changes presents a technical challenge, as current methodologies require tissue fixation that precludes temporal analysis.

The requirement for rapid humidity detection has likely shaped the evolution of hygrosensilla across insect species, resulting in diverse structural adaptations that balance sensitivity with response time [14,15]. In cockroaches, “sensilla capitulum” are shielded by bristles and a cuticular wall, reducing their exposure to air pressure changes [49,50]. Conversely, stick insects have a more exposed “peg-in-pit” structure surrounded by a protective collar, allowing direct environmental interaction [51]. These adaptations suggest species-specific optimisation between exposure and protection.

In *D. melanogaster*, the location of hygrosensilla within the sacculus presents an intriguing paradox. This invagination on the posterior antenna creates a protected environment but may also limit the diffusion of air and potentially slow sensory responses. We propose that passive diffusion of air alone may be insufficient to support the observed rapid behavioural responses to humidity changes. We suggest that active airflow within the sacculus, potentially facilitated by antennal movements, could enhance humidity detection. Although speculative, this concept parallels observations in species such as cockroaches, where antennal fanning enhances olfactory sensitivity, and may warrant investigation in *D. melanogaster* [52,53]. This model would reconcile the apparently contradictory requirements for protection and rapid response.

The chamber-specific structural differences we observe may further enhance this active sensing system. The sharper tapering of sensilla under different humidity conditions could create localized regions of enhanced mechanical sensitivity, optimising detection despite the constraints of the sacculus environment. This specialised architecture, combined with the proposed active airflow mechanism, could enable the rapid and precise humidity detection necessary for adaptive behaviour. Future studies combining structural analysis with real-time measurements of sensilla dynamics could provide valuable insights into how these spatial and temporal aspects of humidity sensing are integrated.

### Concluding remarks

This study provides the first three-dimensional ultrastructural analysis comparing insect hygrosensilla under different humidity conditions. Our integrated approach combining environmental control, rapid preservation, high-resolution imaging, and machine learning-based segmentation establishes a technical framework applicable to studying structural changes in other sensory organs. Key questions remain regarding the temporal dynamics of sensilla structural changes, the molecular mechanisms underlying observed structural differences, and how evolutionary pressures have shaped hygrosensilla architecture across species. Future studies combining structural analysis with real-time measurements could provide valuable insights into the integration of spatial and temporal aspects of humidity sensing.

## Supporting information

S1 Fig Representative high-magnification images of sensilla from chambers 1–3 under high- and low-humidity conditions. (TIF)
