# Supplementary figures and images for: Three-dimensional ultrastructural characterization of Drosophila melanogaster hygrosensilla across humidity conditions
Source: PLoS One. 2025 Sep 29;20(9):e0314841. doi: 10.1371/journal.pone.0314841 (PMC12478899; doi:10.1371/journal.pone.0314841)

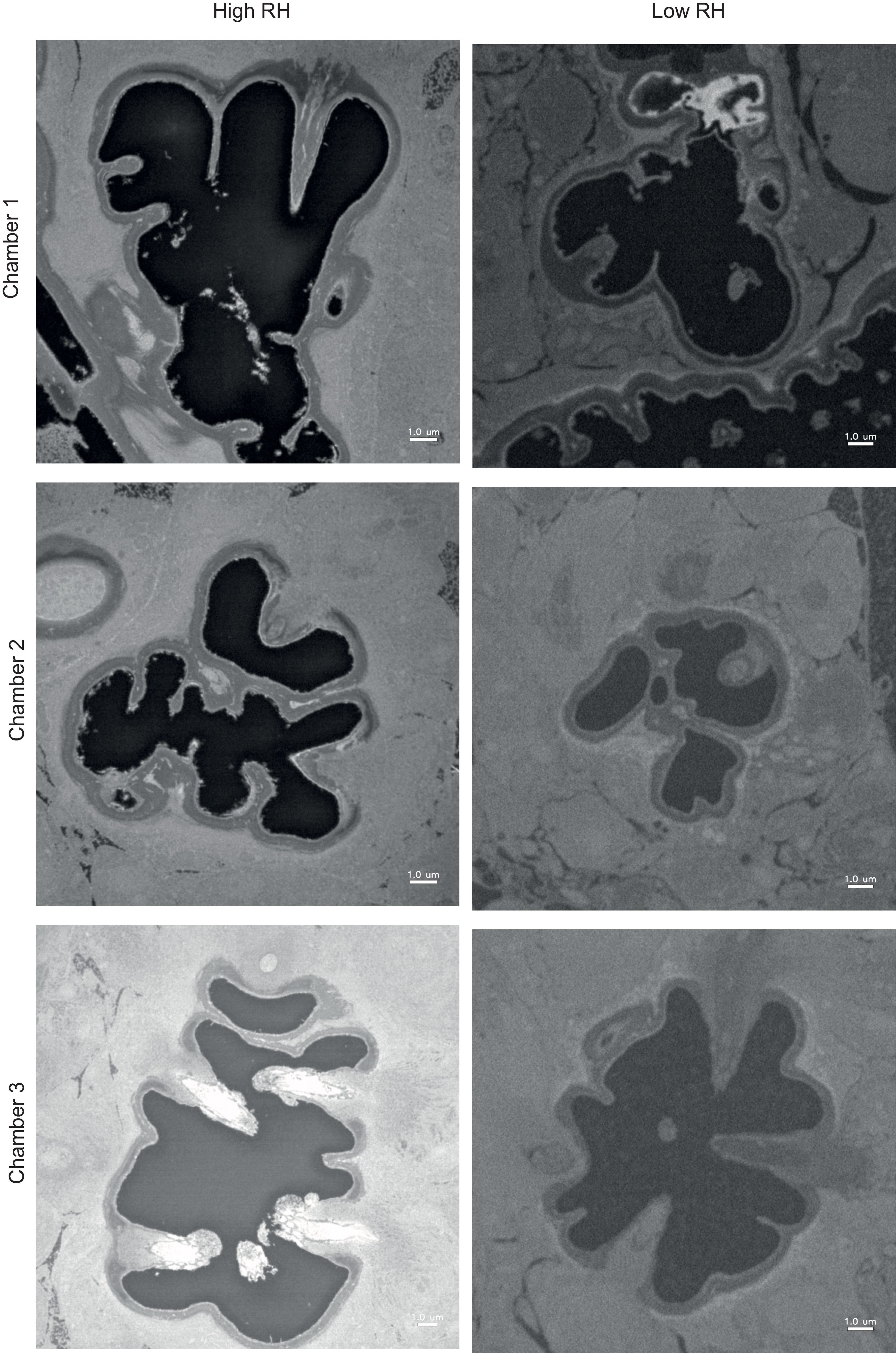

Supplement: S1 Fig — (TIF) [file pone.0314841.s001.tif]
